# Synthesis of Amino Acid Schiff Base Nickel (II) Complexes as Potential Anticancer Drugs *In Vitro*

**DOI:** 10.1155/2020/8834859

**Published:** 2020-09-29

**Authors:** Yang Li, Jianfang Dong, Peiran Zhao, Ping Hu, Dawei Yang, Lei Gao, Lianzhi Li

**Affiliations:** ^1^Zhong Yuan Academy of Biological Medicine, Liaocheng People's Hospital, Liaocheng 252000, China; ^2^Department of Material Science, Shandong Polytechnic Technician College, Liaocheng 252000, China; ^3^School of Chemistry and Chemical Engineering, Liaocheng University, Liaocheng 252000, China

## Abstract

Three hexacoordinated octahedral nickel (II) complexes, [Ni (Trp-sal) (phen) (CH_3_OH)] (1), [Ni (Trp-*o*-van) (phen) (CH_3_OH)]•2CH_3_OH (2), and [Ni (Trp-naph) (phen) (CH_3_OH)] (3) (where Trp-sal = Schiff base derived from tryptophan and salicylaldehyde, Trp-*o*-van = Schiff base derived from tryptophan and *o*-vanillin, Trp-naph = Schiff base derived from tryptophan and 2-hydroxy-1-naphthaldehyde, phen = 1, 10-phenanthroline), have been synthesized and characterized as potential anticancer agents. Details of structural study of these complexes using single-crystal X-ray crystallography showed that distorted octahedral environment around nickel (II) ion has been satisfied by three nitrogen atoms and three oxygen atoms. All these complexes displayed moderate cytotoxicity toward esophageal cancer cell line Eca-109 with the IC_50_ values of 23.95 ± 2.54 *μ*M for 1, 18.14 ± 2.39 *μ*M for 2, and 21.89 ± 3.19 *μ*M for 3. Antitumor mechanism studies showed that complex 2 can increase the autophagy, reactive oxygen species (ROS) levels, and decrease the mitochondrial membrane potential remarkably in a dose-dependent manner in the Eca-109 cells. Complex 2 can cause cell cycle arrest in the G2/M phase. Additionally, complex 2 can regulate the Bcl-2 family and autophagy-related proteins.

## 1. Introduction

The significant findings that cisplatin serves as a chemotherapeutic agent quickly promoted significant breakthroughs in metal-based anticancer complexes [1]. Recently, cisplatin and its analogs such as carboplatin and oxaliplatin are still used worldwide for the treatment of different cancers [2, 3]. However, these drugs exhibit serious side effects including severe toxicity and acquired drug resistance which have limited its clinical applications [4–6]. Therefore, exploring new metal drugs to overcome these limitations is crucial for the treatment of cancer. With this aim, various classes of metal compounds have been synthesized, and their anticancer activity has been successfully evaluated both *in vitro* and *in vivo* [7–9].

Amino acid Schiff base is usually a compound formed by the condensation of amino acid and different active carbonyl groups. In fact, Schiff base compounds and their metal complexes are outstanding in the field of metal-based drugs [10–13]. The Schiff base complexes have been extensively studied in this perspective due to their wide applications in medicine [14–17]. Nickel, as an essential trace element for human beings, animals, microorganisms, and plants, has been known as a component in many enzymes, playing a crucial role in important metabolic reactions [18–23]. In addition to this, various nickel (II) complexes have been synthesized and evaluated as metal-based drugs which demonstrated many bioactivities such as anticonvulsant, antibacterial, antifungal, antimicrobial, antioxidant, and anticancer [24–30].

In our previous work, we investigated the DNA interaction and SOD activity of nickel (II) complexes containing L-phenylalanine Schiff base and 1, 10-phenanthroline [31]. The results showed that three synthesized complexes have the ability to interact with DNA, mainly in the binding and cleaving. Moreover, these complexes also demonstrated promising superoxide scavenging activities which indicate that the nickel (II) complexes could be employed as antiaging agents. In this paper, a series of tryptophan Schiff base nickel (II) complexes have been synthesized and characterized as potential anticancer agents. The *in vitro* antiproliferative activity of complexes 1–3 was investigated by cytotoxicity against four cancer cell lines (MCF-7, SGC-7901, Eca-109, and HepG2), as well as a nontumorigenic cell line HSF. To elucidate the possible anticancer mechanisms, the impacts of complex 2 toward Eca-109 were evaluated for cell morphology (AO/EB, and Hoechst 33342 staining), apoptosis, autophagy, reactive oxygen species, mitochondrial membrane potential, cell cycle arrest, and related protein expression level.

## 2. Results and Discussion

### 2.1. Syntheses of the Complexes and Ligands

**Scheme 1:**
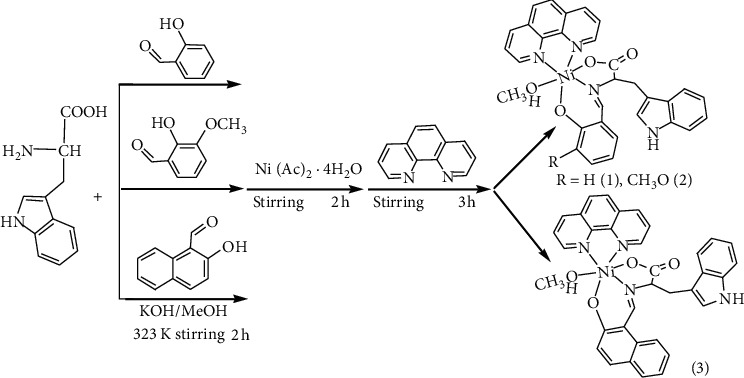
Synthetic routes for the preparation of the complexes [Ni (Trp-sal) (phen) (CH_3_OH)] (1), [Ni (Trp-(o)-van) (phen) (CH_3_OH)]•2CH_3_OH (2), and [Ni (Trp-naph) (phen) (CH_3_OH)] (3).

The complexes 1, 2, and 3 were synthesized in a one-pot method as depicted in Scheme 1. Schiff base L1 (Trp-sal), L2 (Trp-*o*-van), and L3 (Trp-naph) were synthesized according to the procedure reported in the literature [32–34]. Complexes 1, 2, and 3 were characterized using single-crystal X-ray crystallography, element analysis, and IR spectrum. The detailed synthesis procedure is shown in Scheme 1.

### 2.2. Crystal Structure of Three Nickel (II) Complexes

**Figure 1:**
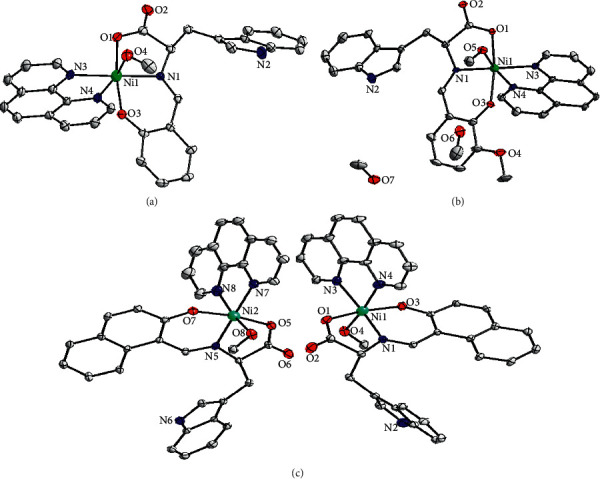
Molecular structures of complexes 1 (a), 2 (b), and 3 (c) with some atoms labeled. Hydrogen atoms on the ligands are omitted for clarity.

As shown in Figure 1, complexes 1 and 2 both have one independent molecular monomer in their asymmetric units, respectively, and two independent molecules in complex 3 in their asymmetric units. Their selected bond distances and angles are listed in Table 1.

Single-crystal X-ray diffraction reveals that Ni (II) atom is six-coordinated by two N atoms from phen ligand, one N atom and two O atoms from Schiff base ligand, and one O atom from coordinated CH_3_OH ligand, forming a distorted octahedral geometric configuration in the structure of complex 1. The equatorial coordination to Ni (II) ion is provided by N3 atom from phen ligand and O1, N1, and O3 atoms from Schiff base ligand (Figure 1(a)). The Ni1 ion lies 0.0168 (22) Å above the equatorial plane. The dihedral angle formed by the equatorial plane and phen ligand is 89.86 (12)°, indicating that they are almost in a vertical position. The two axial sites of the octahedron are occupied by N4 atom from the phen ligand and one O4 atom from the methanol molecule. The N4–Ni–O4 bond angle is less than 180° and is equal to 169.71 (18)°. The Ni–O bond distances vary from 2.009 (4) to 2.132 (4) Å and Ni–N bond lengths range from 2.008 (4) to 2.129 (4) Å. Similar to complex 1, the coordination environment of the Ni1 ion in complex 2 is also a distorted octahedral geometric configuration (Figure 1(b)). The Ni1 ion lies 0.0225 (31) Å above the equatorial plane formed by O1, O3, N1, and N3 atoms. The dihedral angle formed by the equatorial plane and phen ligand is 84.21 (16)°. The bond angle O5–Ni1–N4 = 170.5 (2)° is not close to 180° with the deviation from linearity of 9.5°, which is consistent with the data obtained for the complex 1. Complex 3 crystallized in the monoclinic system, P21 space group, contains two molecules in the asymmetric unit. As shown in Figure 1(c), the coordination geometry of the Ni1 and Ni2 is a slightly distorted octahedron with the small differences in the structure parameters. In the coordination sphere for the Ni1 ion, the equatorial positions are occupied by N1, O1, O3, and N3 atoms from Schiff base ligand and phen ligand, while the two axial sites are occupied by the O4 and N4 atoms. The Ni1 ion lies 0.0438 (31) Å above the equatorial plane with Ni1–N4 = 2.110 (6) Å and Ni1–O4 = 2.126 (5) Å. The phenanthroline ligand coordinated to Ni1 in the direction almost vertical to the equatorial plane with the dihedral angle 88.94 (19)°. Around the Ni2 ion, N8 and O8 atoms occupy the axial positions of the octahedral geometry with the bond angle O8–Ni1–N8 = 167.0 (3)°. The equatorial plane of the octahedron is formed by O5, O7, N5, and N7 atoms. The Ni2 ion is situated 0.0315 (33) Å above the equatorial plane with bond lengths Ni2–N8 = 2.093 (8) Å and Ni2–O8 = 2.121 (5) Å, respectively. The dihedral angle formed by the equatorial plane and the phenanthroline ligand is 85.68 (17)°, which is markedly less than the above dihedral angle, indicating the obviously distorted coordination geometry.

### 2.3. Cytotoxicity Assay *In Vitro*

The cytotoxic activities of the ligands (L1, L2, and L3) and three hexacoordinated octahedral nickel (II) complexes 1–3 and Ni (CH_3_COO)_2_ were evaluated against four human cancer cell lines (including breast cancer MCF-7, gastric cancer SGC-7901, esophageal cancer Eca-109, and hepatocellular carcinoma HepG2) as well as normal human cells HSF using MTT assay method. The cytotoxicity of cisplatin was also evaluated for comparison. As shown in Table 2, Schiff base ligands L1 and L2 showed no toxicity with IC_50_ values being higher than 80 *μ*M to four human cancer cell lines. L3 and Ni (CH_3_COO)_2_ had weak cytotoxic effects toward SGC-7901 and Eca-109 cells with IC_50_ values of 42.70 ± 1.13, 48.36 ± 4.99, and 41.35 ± 0.87, 43.51 ± 1.34, respectively. In contrast, complexes showed moderate cytotoxic toward Eca-109 cells with the IC_50_ values of 23.95 ± 2.54, 18.14 ± 2.39, and 21.89 ± 3.19 *μ*M, respectively. Since the complexes displayed sensitive cytotoxicity toward Eca-109 cells, this cell line was employed for further investigation to explain the anticancer mechanism.

**Figure 2:**
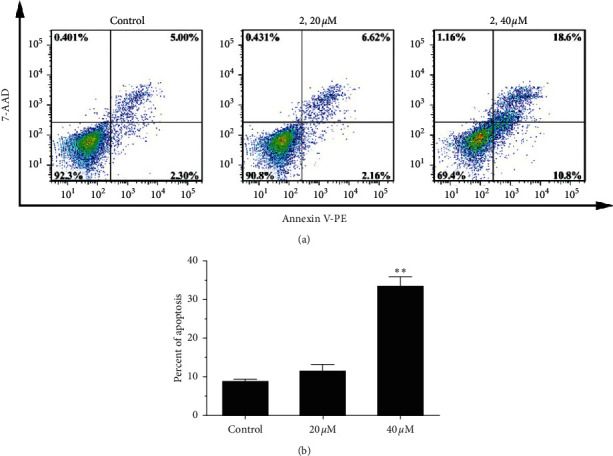
(a) Apoptosis of Eca-109 cells was detected by Annexin V-PE/7-AAD staining assay after coincubation with different concentrations (0, 20, and 40 *μ*M) of complex 2 for 24 h. (b) Quantitative data analysis for the number of cells (% of total) in apoptosis for different treatment groups. Data were presented as mean ± SD (*n* = 3), Student's *t*-test, ^*∗∗*^*P* < 0.01.

Annexin V-PE/7-AAD staining assay was performed to analyze the cell death induced by complex 2 for 24 h using flow cytometry.  Figure 2 showed that the percentage of apoptotic cells, which were 7.30% for control, 8.78% for 20 *μ*M, and 29.40% for 40 *μ*M, had increased remarkably in a dosage-dependent manner.

### 2.4. Morphological Analysis of Cells by AO/EB and Hoechst 33342 Staining

**Figure 3:**
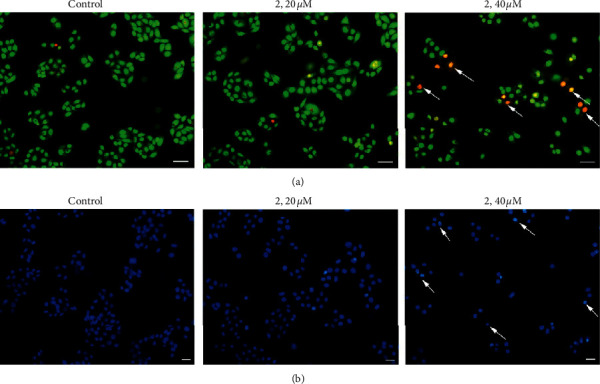
Eca-109 cells were stained with AO/EB (a) and Hoechst 33342 (b) after being exposed to complex 2 (0, 20, and 40 *μ*M) for 24 h under a fluorescence microscope. Representative photomicrographs from three independent experiments. Arrows indicate apoptotic bodies. Scale bars: 20 *µ*m.

Apoptosis and necrosis, as the common cellular responses of anticancer complexes toward cancer cells, always induced the morphological changes of cells [35]. Acridine orange (AO)/ethidium bromide (EB) are used together to differentiate between viable, apoptotic, and necrotic cells, where live cells display bright green fluorescence with normal cytoplasm and nuclei morphology and early apoptotic cells display green fluorescence with nuclear shrinkage and chromatin condensation. Meanwhile, necrotic cells show red fluorescing without chromatin fragmentation, and late apoptotic cells stain red fluorescence with nuclear shrinkage and chromatin condensation [36]. As shown in Figure 3(a), the untreated Eca-109 cells were stained with uniform green fluorescence. After the treatment with 20 *μ*M and 40 *μ*M complex 2 for 24 h, the obvious morphological changes such as nuclear shrinkage, chromatin condensation, and late apoptotic cells containing red apoptotic bodies as the arrow pointed were observed. The apoptosis was also investigated with the Hoechst 33342 staining method. Hoechst 33342 is a specific stain for nuclei in living cells that had a light green fluorescent cytoplasm and apoptotic cells had bright, fragmented nuclei containing condensed chromatin. As indicated in Figure 3(b), after the treatment with 20 *μ*M and 40 *μ*M complex 2 for 24 h, the apoptotic characteristics were also observed, especially for 40 *μ*M. These results demonstrated that complex 2 can induce the apoptosis of Eca-190 cells in a concentration-dependent manner.

### 2.5. Intracellular Reactive Oxygen Species (ROS) Levels' Determination

**Figure 4:**
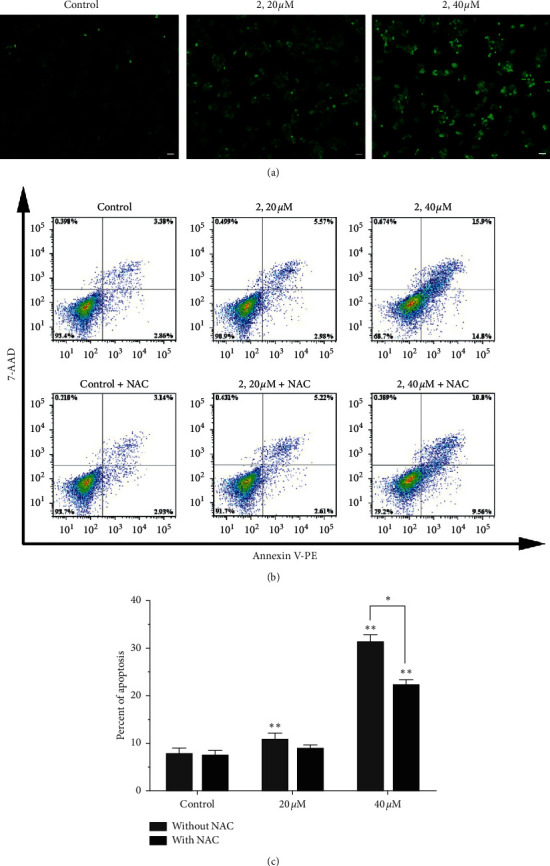
(a) Intracellular ROS was detected in Eca-190 cells after exposure to complex 2 (0, 20, and 40 *μ*M) for 24 h under a fluorescence microscope. Representative photomicrographs from three independent experiments. Scale bars: 20 *µ*m. (b) Apoptosis of Eca-109 cells was detected by flow cytometry after treated with different concentrations (0, 20, and 40 *μ*M) of complex 2 for 24 h with or without NAC pretreatment. (c) Quantitative data analysis for the number of cells (% of total) in apoptosis for different treatment groups. Data were presented as mean ± SD (*n* = 3), Student's *t*-test, ^*∗*^*P* < 0.05, ^*∗∗*^*P* < 0.01.

It has been reported that the increasing intracellular ROS levels could result in mitochondrial dysfunction, enter the nucleus to cause DNA damage, and finally induce cell apoptosis [37]. In order to elucidate the association between cytotoxicity and the generation of ROS, the Eca-109 cells were exposed to 20 *μ*M and 40 *μ*M complex 2 for 24 h, and then the ROS levels were evaluated using a 2',7'-dichlorodihydrofluorescein diacetate (DCFH-DA) as a fluorescent probe. The DCFH-DA dye can be cleaved by intracellular esterases into its nonfluorescent form 2',7'-dichloro-3,6-fluorandiol (DCFH). Then, the no-fluorescent substrate is oxidized by intracellular free radicals to produce a fluorescent product, dichlorofluorescein (DCF) [38]. As shown in Figure 4(a), in the control group, low intracellular fluorescence intensity can be observed due to the low ROS level, which is difficult for DCHF-DA to be transferred into the fluorescent product DCF. After the treatment of Eac-109 cells with 20 *μ*M and 40 *μ*M complex 2 for 24 h, numerous green fluorescent points can be found in the cells, and with the increasing concentration of complex 2, a more bright green fluorescent point can be observed, which means the increase of ROS level. These results demonstrated that complex 2 could increase the intracellular ROS level in a dosage-dependent manner.

N-acetyl-L-cysteine (NAC), as a ROS scavenger, was used to examine the role of ROS generation in complex 2 induced apoptotic cell death. The percentage of apoptotic cells was detected by Annexin V-PE/7-AAD staining assay after complex 2 treatment with or without NAC. With the NAC pretreatment, the percentage of apoptosis cells induced by complex 2 was reduced as shown in Figures 4(b) and 4(c). Those results suggested that ROS generated by complex 2 plays an important role in inducing apoptotic in Eca-109 cells.

### 2.6. Autophagy Induced by Complex 2

**Figure 5:**
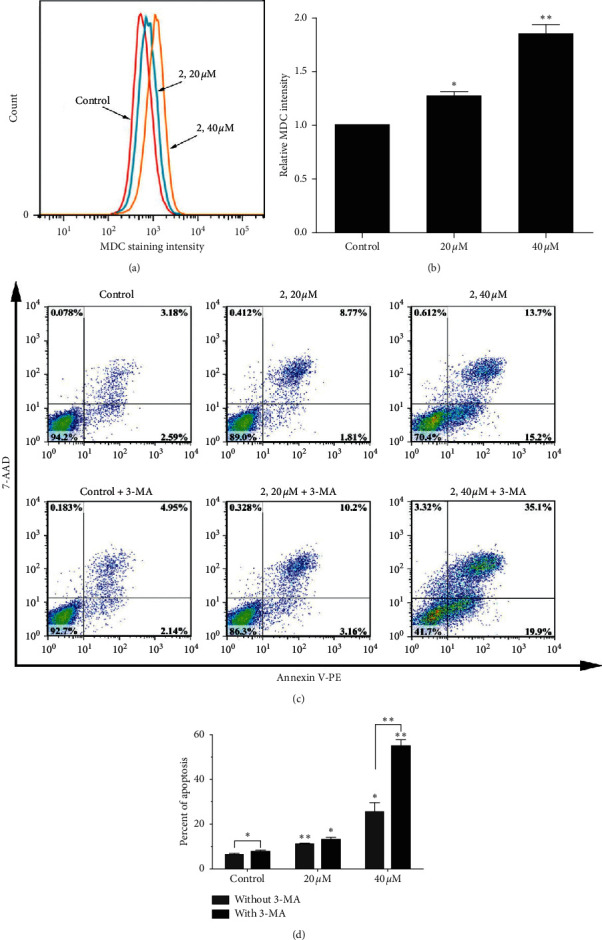
(a) Autophagy was detected in Eca-190 cells after exposure to complex 2 (0, 20, and 40 *μ*M) for 24 h using MDC staining assay. (b) Relative quantitative data analysis of MDC fluorescence intensity for different treatment groups. (c) Apoptosis of Eca-109 cells was detected by flow cytometry after treated with complex 2 (0, 20, and 40 *μ*M) for 24 h with or without pretreatment of 3-MA. (d) Quantitative data analysis for the number of cells (% of total) in apoptosis for different treatment groups. Data were presented as mean ± SD (*n* = 3), Student's *t*-test, ^*∗*^*P* < 0.05, ^*∗∗*^*P* < 0.01.

Autophagy, as a life phenomenon and lysosomal degradation pathway, exists widely in the cell, and it is crucial for homeostasis under normal conditions [39]. Autophagy has been considered to be a survival response to growth factor or nutrient deprivation and also reported to play a critical role in tumor cell suppression [40]. Monodansylcadaverine (MDC), a ﬂuorescent compound, is incorporated into multilamellar bodies by both an ion trapping mechanism and the interaction with membrane lipids, as a probe for the detection of autophagic vacuoles in cultured cells. In order to determine the autophagic effect of the complex 2 on Eca-190 cells, the cells were treated with complex 2 for 24 h and then stained with MDC. As shown in Figures 5(a) and 5(b), the MDR staining intensity increased significantly after treatment with 20 *μ*M and 40 *μ*M complex 2 compared to the control group. These results indicated that complex 2 could increase the autophagy level of Eca-190 cells in a dosage-dependent manner.

To investigate whether the autophagy affects the cell viability, Eca-109 cells were treated by different concentrations of the complex 2 with or without autophagic inhibitor 3-methyladenine (3-MA). As shown in Figures 5(c) and 5(d), 3-MA had nontoxicity toward cells, but 3-MA decreased the cell viability caused by complex 2 in various degrees, which indicates that the autophagy inhibits the cell death.

### 2.7. Mitochondrial Dysfunction Induced by Complex 2

**Figure 6:**
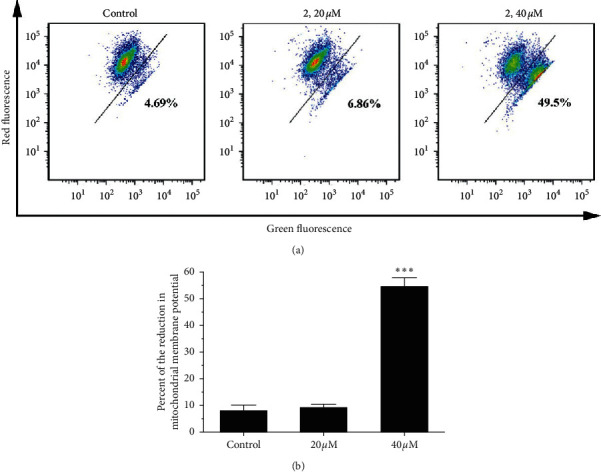
(a) Eca-109 cells' mitochondrial membrane potential was detected by JC-1 staining assay after coincubation with different concentrations (0, 20, and 40 *μ*M) of complex 2 for 24 h. (b) Quantitative data analysis for the number of cells (% of total) in the reduction of mitochondrial membrane potential for different treatment groups. Data were presented as mean ± SD (*n* = 3), Student's *t*-test, ^*∗∗*^*P* < 0.01.

As an important role in apoptosis, mitochondria can release proapoptotic factors such as cytochrome *c* and other apoptosis-inducing factors [41]. The mitochondrial dysfunction associated with apoptosis was assayed using 5,5′,6, 6′-tetrachloro-1,1′, 3,3′-tetraethylbenzimidalylcarbo cyanine iodide (JC-1) as a fluorescent probe. JC-1 forms aggregates and emits a red fluorescence corresponding to high mitochondrial membrane potential [42, 43]. After mitochondrial dysfunction, the mitochondrial membrane potential (MMP) will be decreased, and JC-1 forms monomer and emits green fluorescence. As shown in Figure 6, after 24 h of incubation with complex 2, the green fluorescence of the JC-1 monomers increases from 4.69% (control) to 6.86% (20 *μ*M) and 49.5% (40 *μ*M), indicating loss of MMP.

### 2.8. Cell Cycle Arrest Assay

**Figure 7:**
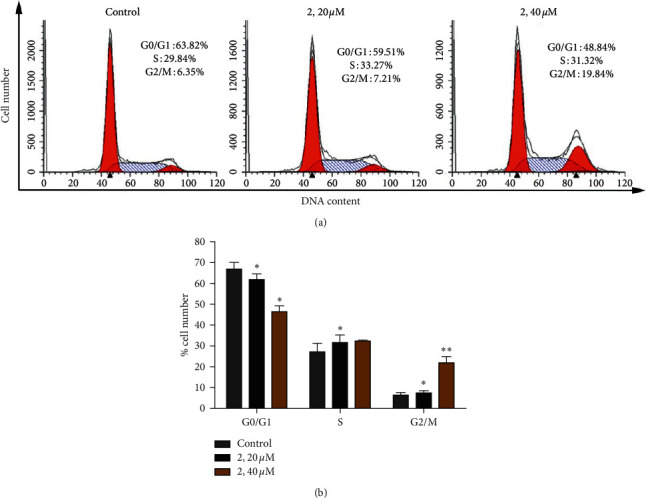
(a) Cell cycle distribution was performed by PI staining after being coincubated with complex 2 (0, 20, and 40 *μ*M) for 24 h. (b) Quantitative data analysis for the number of cells (% of total) in each cell phase for different treatment groups. Data were presented as mean ± SD (*n* = 3), Student's *t*-test, ^*∗*^*P* < 0.05, ^*∗∗*^*P* < 0.01.

It is reported that inhibition of cancer cell proliferation by cytotoxic drugs could be the result of cell cycle arrest, apoptosis, or their combination. To investigate whether the antiproliferative effect of complex 2 on Eca-109 cells was triggered by cell cycle arrest, the cell cycle phase ratio was measured by flow cytometry with propidium iodide (PI) staining. As shown in Figure 7, in the control, the percentage in the cell at the G2/M phase is 6.35%. After the cells were coincubated with different concentrations of complex 2, the percentage in the cell at the G2/M phase is 7.21% for 20 *μ*M and 19.84% for 40 *μ*M. Meanwhile, the percentage in the cell at the G0/G1 phase is 63.82% for control, 59.51% for 20 *μ*M, and 48.84% for 40 *μ*M, respectively. Obviously, these results demonstrate that complex 2 inhibits cell growth in Eca-109 at the G2/M phase.

### 2.9. The Expression of Bcl-2 Family and Autophagy-Related Proteins

**Figure 8:**
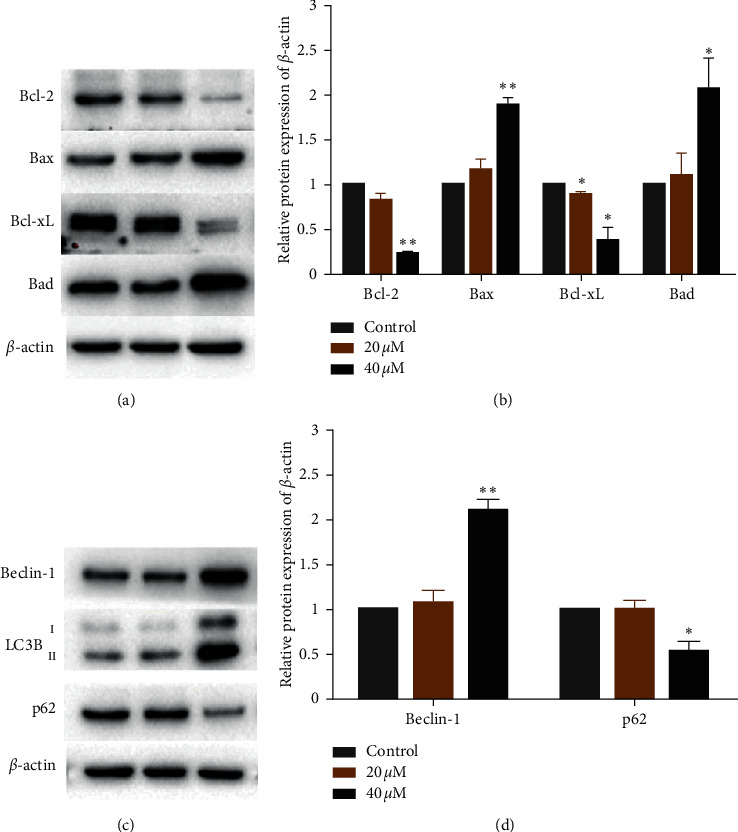
The expression of (a) Bcl-2 family and (c) autophagy-related proteins in Eca-109 cells treated with complex 2 for 24 h. (b, d) Quantitative analysis of Western blotting from (a) and (c) by Image Lab program with *β*-actin as the internal control. Data were presented as mean ± SD (*n* = 3), Student's *t*-test, ^*∗*^*P* < 0.05, ^*∗∗*^*P* < 0.01.

Bcl-2 family proteins, known as one important regulatory factor of apoptosis, were investigated in this study by Western blot assay [35, 44]. As shown in Figures 8(a) and 8(b), the expression levels of antiapoptotic proteins Bcl-2 and Bcl-xL were significantly decreased, whereas proapoptotic proteins Bax and Bad were significantly increased in a concentration-dependent manner after Eca-109 cells being exposed to the complex 2 for 24 h. These results suggested that complex 2 can induce apoptotic in the mitochondrial pathways.

Autophagy, as a cellular degradation process, can be activated in tumor cells during anticancer drugs action. With the initiation of autophagy and the formation of autophagosome, Beclin-1 binds LC3B and interacts with the ubiquitin-binding protein p62. As shown in Figures 8(c) and 8(d), the expression levels Beclin-1 and LC3B-II increased, whereas p62 decreased in a concentration-dependent manner after Eca-109 cells being exposed to the complex 2 for 24 h. These results suggested autophagy can be activated during complex 2 anticancer actions.

## 3. Conclusions

Three hexacoordinated octahedral nickel (II) complexes were synthesized and characterized as potential anticancer agents in this study. These complexes showed moderate cytotoxicity toward Eca-109 cells and complex 2 was selected for further investigation to explain the mechanism of apoptosis. The percentage of apoptotic cells was increased remarkably in a dose-dependent manner after coincubation with complex 2 for 24 h. AO/EB and Hoechst 33342 staining showed that complex 2 can change the morphological of cells and induce apoptosis in a concentration-dependent manner. Complex 2 can increase the intracellular ROS level and induce a decrease in the mitochondrial membrane potential, which plays an important role in inducing apoptotic. The cell cycle arrest studies demonstrate that complex 2 inhibits cell growth in Eca-109 cells at the G2/M phase. The autophagy level could be also increased by complex 2, and autophagy, playing a protective role, inhibits cell death. Additionally, complex 2 can regulate the Bcl-2 family and autophagy-related proteins in a concentration-dependent manner. In summary, we found that amino acid Schiff base nickel complexes can effectively inhibit cancer cell growth mainly through mitochondrial dysfunction, intracellular ROS accumulation, and ROS- mediated DNA damage, and this work will be helpful for the design and synthesis of new amino acid Schiff base nickel complexes as promising antitumor agents.

## 4. Experimental

### 4.1. Materials and Methods

Salicylaldehyde, *o*-vanillin, and 2-hydroxy-1-naphthaldehyde were purchased from Alfa Aesar. D-tryptophan was obtained from Beijing Jingke Company. The other chemicals were obtained from commercial sources and used without further purification unless otherwise noted. All reagents were AR grade or biochemical quality. Ultrapure Milli-Q water was used in all experiments.

Elemental analyses (*C*, *H*, *N*) were performed on a Perkin-Elmer 2400 II analyzer, and IR spectra were recorded as KBr pellets on a Nicolet 5700 FT-IR instrument in the frequency range 400–4000 cm^−1^.

### 4.2. Syntheses of the Complexes

#### 4.2.1. Synthesis of Complex [Ni (Trp-sal) (phen) (CH_3_OH)] (1)

For complex 1, D-tryptophan (1 mmol, 204.2 mg) and potassium hydroxide (1 mmol, 56.1 mg) were dissolved in methanol (15 mL) at 50°C. A methanol solution (3 mL) of salicylaldehyde (1 mmol, 0.11 mL) was added and stirred for 2 h. Subsequently, an aqueous solution (3 mL) of nickel acetate tetrahydrate (248.86 mg, 1 mmol) was added dropwise and stirred for 2 h. Finally, a methanol solution (5 mL) of 1,10-phenanthroline (1 mmol, 198.2 mg) was added and continuously stirred for 3 h. After the reaction was completed, the resultant solution was held at room temperature for weeks, whereupon green blocky crystals suitable for X-ray diffraction were obtained. Yield: 82%. Anal. Found (%) for C_31_H_26_N_4_NiO_4_ (Mol. wt = 577.27): C, 64.15%; H, 4.86%; N, 9.35%. Calculated (%) for C, 64.50%; H, 4.54%; N, 9.70%. IR spectra of the complex 1 were shown in Figure S1(a). Selected IR bands (KBr pellets,/cm^−1^, s, strong absorption; *m*, middle absorption; *w*, weak absorption): 3,411 (s), 1,636 (s), 1,595 (s), 1,516 (m), 1,496 (m), 1,467 (m), 1,426 (m), 1,339 (m), 1,305 (m), 1,223 (m), 1,182 (w), 1,127 (w), 1,085 (w), 1,011 (w), 962 (m), 848 (m), 765 (w), 728 (m), 642 (w), 545 (w), 451 (w), 427 (w).

#### 4.2.2. Synthesis of the Complex [Ni (Trp-o-van) (phen) (CH3OH)]•2CH3OH (2)

The preparation of complex 2 follows the same procedure as that of complex 1 except that a solution of *o*-vanillin (1 mmol, 152.2 mg) in methanol (3 mL) was used to take the place of salicylaldehyde solution. Green single crystals of 2 suitable for X-ray diffraction were obtained at room temperature. Yield: 85%. Anal. Found (%) for C_34_H_36_N_4_NiO_7_ (Mol. wt = 671.38): C, 60.48%; H, 5.84%; N, 8.57%. Calculated (%) for C, 60.82%; H, 5.40%; N, 8.34%. IR spectra of the complex 2 were shown in Figure S1(b). Selected IR bands (KBr pellets,/cm^−1^, s, strong absorption; *m*, middle absorption; *w*, weak absorption): 3,404 (s), 1,634 (s), 1,596 (s), 1,517 (m), 1,495 (m), 1,469 (m), 1,443 (m), 1,426 (m), 1,384 (m), 1,287 (m), 1,216 (m), 1,127 (w), 1,083 (w), 1,010 (w), 988 (m), 850 (m), 744 (w), 728 (m), 643 (w), 535 (w), 426 (w).

#### 4.2.3. Synthesis of the Complex [Ni (Trp-Naph) (phen) (CH3OH)] (3)

The preparation of complex 3 follows the same procedure as that of complex 1 except that a solution of 2-Hydroxy-1-naphthaldehyde (1 mmol, 172.2 mg) in methanol (3 mL) was used to take the place of salicylaldehyde solution. Green crystals of 3 suitable for X-ray diffraction were also obtained at room temperature. Yield: 80%. Anal. Found (%) for C_35_H_28_N_4_NiO_4_ (Mol. wt = 627.32): C, 67.38%; H, 4.35%; N, 9.21%. Calculated (%) for C, 67.01%; H, 4.50%; N, 8.93%. IR spectra of the complex 3 were shown in Figure S1(c). Selected IR bands (KBr pellets,/cm^−1^, s, strong absorption; *m*, middle absorption; *w*, weak absorption): 3,412 (s), 1,620 (s), 1,540 (s), 1,516 (m), 1,458 (m), 1,426 (m), 1,343 (m), 1,302 (m), 1,249 (m), 1,182 (w), 1,127 (w), 1,092 (w), 1,037 (w), 974 (m), 845 (m), 748 (w), 728 (m), 642 (w), 564 (w), 454 (w), 427 (w).

### 4.3. X-Ray Crystallography

The X-ray diffraction data of complexes 1, 2, and 3, were collected at 298 K with Mo-Ka radiation (*λ* = 0.071073 nm) using a Bruker Smart-1000 CCD diffractometer equipped with a graphite monochromator. Diffraction intensities for the complexes were collected by using the *ω*-scan technique. The structures were solved by direct methods with the aid of successive difference Fourier maps using SHELXS-2014 and refined anisotropically by full-matrix least-squares on *F*^2^ using SHELXL-2014 [45]. All nonhydrogen atoms were refined anisotropically. The organic hydrogen atoms were generated geometrically and allowed to refine using a riding model. Hydrogen atoms attached to carbon were placed in calculated positions and refined using a riding model with isotropic displacement parameters 1.2 or 1.5 times the isotropic equivalent of their carrier atoms. Crystallographic data and experimental details for structural analyses of 1, 2, and 3 are summarized in Table 3. Crystallographic data for the three complexes have been deposited with the Cambridge Crystallographic Data Centre as supplementary publication CCDC reference numbers 1841712, 1829802, and 1829801, respectively. Any inquiries related to the data can be e-mailed to deposit@ccdc.cam.ac.uk.

### 4.4. Cell Culture

#### 4.4.1. Cytotoxicity *In Vitro* Assay

3-(4,5-Dimethylthiazol-2-yl)-2, 5-diphenyltetrazolium bromide (MTT) method was used to assay the cytotoxicity *in vitro*. Complexes 1–3, ligands (L1, L2, and L3), Ni (CH_3_COO)_2_, and cisplatin were dissolved in DMSO at the concentration of 20 mM as a stock solution for subsequent testing. Four different cancer cell lines (MCF-7, SGC-7901, Eca-109, and HepG2) and one normal cell line (HSF) were seeded in 96-well and cultured at 37^o^C in 5% CO_2_. After 24 h, the medium was replaced by different concentrations of complex and incubated for 48 h. 10 *μ*L MTT dye (5 mg/mL) was added to each well and incubated for another 4 h. After that, the medium was removed and DMSO (100 *μ*L) was added to solubilize the MTT formazan. A microplate reader (Bio-Rad iMark™) was used to measure the OD value of each well at a wavelength of 490 nm. Each experiment was repeated at least three times, and GraphPad Prism 5.0 was used to analyze the IC_50_ values.

#### 4.4.2. Apoptosis Assay by Hoechst 33342 Staining

With the density of 2 × 10^5^ cells per well, Eca-109 cells were cultured in a 6-well plate for 24 h. After that, the medium was replaced by different concentrations of complex 2 and incubated for another 24 h. Then, cells were washed twice with ice-cold PBS, stained with 10 *μ*g/mL Hoechst 33342 for 15 minutes, and photographed by fluorescence microscopy (Nikon Eclipse Ti–E, Nikon Instruments Inc., Japan).

#### 4.4.3. Acridine Orange/Ethidium Bromide (AO/EB) Double Staining

AO/EB double staining was used to observe the changes in apoptotic cells. Eca-109 cells were cultured in a 6-well plate for 24 h. Then, cells were treated with different concentrations of complex 2 for another 24 h. After that, cells were washed twice with ice-cold PBS, stained with 100 *μ*g/mL AO/EB for 10 minutes, and photographed by fluorescence microscopy (Nikon Eclipse Ti–E, Nikon Instruments Inc., Japan).

#### 4.4.4. Reactive Oxygen Species (ROS) Levels Studies

Eca-109 cells were seeded into a 6-well plate at a density of 2 × 10^5^ cells per well and incubated for 24 h. Then, the medium was replaced and incubated with different concentrations of complex 2 for another 24 h. After that, cells were washed twice with ice-cold PBS, stained with 10 *μ*M DCFH-DA for 30 minutes, and photographed by fluorescence microscopy (Nikon Eclipse Ti–E, Nikon Instruments Inc., Japan).

N-acetyl-_L_-cysteine (NAC), as a ROS scavenger, was added in a medium for 1 h before the addition of complex 2 to examine the effect of ROS on cell viability. The percentage of apoptotic cells was detected by Annexin V-PE/7-AAD staining assay after complex 2 treatment with or without NAC.

#### 4.4.5. Apoptosis by Flow Cytometry

Different stages of apoptosis induced by complex 2 were determined by Annexin V-PE and 7-AAD staining according to the manufacture's protocol for the Annexin V Apoptosis Detection Kit (BD, Bioscience). Eca-109 cells were seeded in a 6-well plate with a density of 2 × 10^5^ cells per well and cultured at 37°C in 5% CO_2_ for 24 h. Then, different concentrations of complex 2 were added into the above well and incubated for another 24 h. The cells were resuspended in binding buffer, stained with 5 *μ*L Annexin V-PE and 5 *μ*L 7-AAD at room temperature for 15 min, and analyzed using the flow cytometer (BD FACS Aria III).

#### 4.4.6. Autophagy Assay

Eca-109 cells were seeded in 6-well plates and incubated at 37^o^C in 5% CO_2_ for 24 h. Then, the medium was replaced with different concentrations of complex 2 for another 24 h. After that, the medium was removed and the cells were washed twice with ice-cold PBS. Then, the cells were stained with MDC (monodansylcadaverine) solution (50 *μ*M) for 15 min, and the data were obtained by flow cytometry (BD FACS Aria III).

3-MA, as an autophagic inhibitor, was added in a medium for 1 h before the addition of complex 2 to examine the effect of autophagy on cell viability. The percentage of apoptotic cells was detected by Annexin V-PE/7-AAD staining assay after complex 2 treatment with or without 3-MA.

#### 4.4.7. Mitochondrial Membrane Potential Measurement

After being treated with different concentrations of complex 2, the cells were collected and incubated in 500 *μ*L PBS containing 10 *μ*g/mL JC-1 for 20 min at 37°C in the dark. Then, the cells were resuspended in PBS and analyzed by flow cytometry (BD FACS Aria III).

#### 4.4.8. Cell Cycle Arrest Assay

After treatment with different concentrations of complex 2, the cells were collected and fixed with 70% ethanol for the night. Then, the cells were incubated in 500 *μ*L PBS containing Triton X-100 (0.1% v/v), RNase A (0.2 mg/mL), and propidium iodide (PI, 0.02 mg/mL) for 15 min. Then, the cells were analyzed by flow cytometry (BD FACS Aria III).

#### 4.4.9. Western Blot Assay

After incubation with different concentrations of complex 2, cells were harvested in a radioimmunoprecipitation assay (RIPA) buffer with protease inhibitors and phosphatase inhibitors. BCA Protein Assay Kit was used to determine the protein concentration. Then, the expression levels of proteins associated with Bcl-2 family and autophagy were measured by Western blot assay.

### 4.5. Statistical Analysis

All experiments were repeated three times. Statistical significance was evaluated by Student's *t*-test, and ^*∗*^ means *P* < 0.05, ^*∗*^^*∗*^ means *P* < 0.01, and ^*∗*^^*∗*^^*∗*^ means *P* < 0.001.

## Figures and Tables

**Table 1:** Selected bond lengths (Å) and bond angles (°) for complexes 1, 2, and 3.

Complex 1					
Ni1-N1	2.008 (4)	Ni1-O3	2.009 (4)	Ni1–O1	2.066 (4)
Ni1–N3	2.082 (4)	Ni1–N4	2.129 (4)	Ni1–O4	2.132 (4)
N1-Ni1-O3	90.71 (17)	N1-Ni1-O1	81.23 (16)	O3-Ni1-O1	171.92 (16)
N1-Ni1-N3	176.51 (19)	O3-Ni1-N3	91.93 (17)	O1-Ni1-N3	96.15 (16)
N1-Ni1-N4	98.65 (17)	O3-Ni1-N4	91.28 (17)	O1-Ni1-N4	90.50 (16)
N3-Ni1-N4	79.02 (17)	N1-Ni1-O4	91.44 (17)	O3-Ni1-O4	86.55 (18)
O1-Ni1-O4	93.05 (18)	N3-Ni1-O4	90.99 (19)	N4-Ni1-O4	169.71 (18)

Complex 2					

Ni1-N1	2.007 (6)	Ni1-O3	2.025 (5)	Ni1-O1	2.075 (5)
Ni1-N3	2.092 (6)	Ni1-N4	2.125 (5)	Ni1-O5	2.130 (4)
N1-Ni1-O3	90.4 (2)	N1-Ni1-O1	81.2 (2)	O3-Ni1-O1	171.42 (19)
N1-Ni1-N3	175.3 (2)	O3-Ni1-N3	92.2 (2)	O1-Ni1-N3	96.3 (2)
N1-Ni1-N4	97.0 (2)	O3-Ni1-N4	87.1 (2)	O1-Ni1-N4	95.74 (19)
N3-Ni1-N4	79.3 (2)	N1-Ni1-O5	92.4 (2)	O3-Ni1-O5	90.99 (19)
O1-Ni1-O5	87.46 (18)	N3-Ni1-O5	91.5 (2)	N4-Ni1-O5	170.5 (2)

Complex 3					

Ni1–N1	1.979 (6)	Ni1–O3	2.005 (5)	Ni1–O1	2.043 (5)
Ni1-N3	2.080 (7)	Ni1-N4	2.110 (6)	Ni1-O4	2.126 (5)
Ni2-N5	1.988 (6)	Ni2-O7	1.997 (5)	Ni2-O5	2.033 (5)
Ni2-N (7)	2.087 (7)	Ni2-N8	2.093 (8)	Ni2-O8	2.121 (5)
N1–Ni1–O3	89.0 (2)	N1–Ni1–O1	81.4 (2)	O3–Ni1–O1	170.3 (2)
N1-Ni1-N3	172.3 (2)	O3-Ni1-N3	98.1 (2)	O1-Ni1-N3	91.3 (2)
N1-Ni1-N4	97.8 (2)	O3-Ni1-N4	89.2 (2)	O1-Ni1-N4	90.3 (2)
N3-Ni1-N4	79.4 (3)	N1-Ni1-O4	93.3 (2)	O3-Ni1-O4	86.3 (2)
O1-Ni1-O4	96.1 (2)	N3-Ni1-O4	90.2 (3)	N4-Ni1-O4	168.0 (2)
N5-Ni2-O7	89.1 (2)	N5-Ni2-O5	80.9 (2)	O7-Ni2-O5	169.8 (2)
N5-Ni2-N7	177.7 (2)	O7-Ni2-N7	92.7 (2)	O5-Ni2-N7	97.3 (2)
N5-Ni2-N8	99.2 (3)	O7-Ni2-N8	88.0 (3)	O5-Ni2-N8	91.7 (2)
N (7)-Ni2-N8	79.3 (3)	N5-Ni2-O8	93.0 (2)	O7-Ni2-O8	87.7 (2)
O5-Ni2-O8	94.7 (2)	N (7)-Ni2-O8	88.6 (3)	N8-Ni2-O8	167.0 (3)

**Table 2:** IC_50_ values (*μ*M) of complexes 1, 2, and 3 against human cell lines.^a^

Complex	MCF-7	SGC-7901	Eca-109	HepG2	HSF
Trp + sal + Ni + phen (1)	75.75 ± 0.41	33.99 ± 2.50	23.95 ± 2.54	>80.00	60.94 ± 1.76
Trp + *o*-van + Ni + phen (2)	16.09 ± 1.32	15.69 ± 1.46	18.14 ± 2.39	47.29 ± 0.35	23.91 ± 0.74
Trp + naph + Ni + phen (3)	23.74 ± 0.55	24.29 ± 0.95	21.89 ± 3.19	28.11 ± 1.34	32.54 ± 1.98
L1	>80.00	>80.00	>80.00	>80.00	——
L2	>80.00	>80.00	>80.00	>80.00	——
L3	>80.00	42.70 ± 1.13	48.36 ± 4.99	>80.00	——
Ni (CH_3_COO)_2_	>80.00	41.35 ± 0.87	43.51 ± 1.34	>80.00	——
Cisplatin	12.34 ± 0.75	11.73 ± 1.45	11.87 ± 0.19	13.94 ± 0.58	17.13 ± 1.31

^a^Cell viability was determined by MTT assay after treatment for 48 h.

**Table 3:** Crystallographic and structure refinement data for complexes 1, 2, and 3.

Complex	1	2	3
Empirical formula	C_31_H_26_N_4_NiO_4_	C_34_H_36_N_4_NiO_7_	C_35_H_28_N_4_NiO_4_
Formula weight	577.27	671.38	627.32
Temperature (K)	298 (2)	298 (2)	298 (2)
Wavelength (Å)	0.71073	0.71073	0.71073
Crystal system	Monoclinic	Monoclinic	Monoclinic
Space group	C 2	P 21/c	P 21
*a* (Å)	16.9956 (14)	12.9281 (12)	12.5532 (11)
*b* (Å)	16.7323 (13)	11.9960 (11)	19.1631 (16)
*c* (Å)	12.4490 (9)	22.131 (2)	14.850 (4)
*α* = *γ* (°)	90	90	90
*β* (°)	130.648 (3)	104.568 (2)	111.483 (3)
*V* (Å^3^)	2686.0 (4)	3321.8 (5)	3324.2 (10)
*Z*	4	4	4
*D*_calc_ (Mg·m^−3^)	1.427	1.342	1.257
*F* (000)	1200	1408	1310
Absorption coefficient (mm^−1^)	0.767	0.637	0.626
Crystal size (mm)	0.22 × 0.21 × 0.15	0.36 × 0.20 × 0.11	0.43 × 0.36 × 0.25
*θ* Range (°)	2.434 to 25.016	2.549 to 25.020	2.587 to 25.020
Limiting indices	–20≤ *h* ≤ 15; –19≤ *k* ≤ 19;	–10≤ *h* ≤ 15; –12≤ *k* ≤ 14;	–14≤ *h* ≤ 11; –22≤ *k* ≤ 21;
	–14*l* ≤ 14	–26*l* ≤ 16	–17*l* ≤ 17
Reflections collected	6750	15812	16814
Unique reflections	4497	5824	10297
*R*_int_	0.0243	0.0988	0.0467
Data, restraints, parameters	4497, 1, 363	5824, 0, 421	10297, 1649, 795
Goodness–of–fit on *F*^2^	0.961	1.089	0.964
Absolute structure parameter	−0.009 (11)		0.000 (15)
Final *R* indices [*I* > 2*σ* (*I*)]	*R*_*1*_ = 0.0381, *wR*_*2*_ = 0.0787	*R*_*1*_ = 0.0949, *wR*_*2*_ = 0.2006	*R*_*1*_ = 0.0502, *wR*_*2*_ = 0.1044
*R* Indices (all data)	*R*_*1*_ = 0.0560, *wR*_*2*_ = 0.0868	*R*_*1*_ = 0.1568, *wR*_*2*_ = 0.2242	*R*_*1*_ = 0.1001, *wR*_*2*_ = 0.1233
Largest diff. Peak and hole (e·Å^−3^)	0.437 and -0.196	0.904, and -0.853	0.295, and -0.281

## Data Availability

The data used to support the findings of this study are included within the article and the supplementary information file.

## Supplementary Materials

Supplementary Materials Figure S1: FTIR spectra of the title complexes 1, 2, and 3. Figure S2: CheckCIF/PLATON reports for the crystal structure determinations of the Complexes 1, 2, and 3.
