# Supplementary material for: Synthesis of Amino Acid Schiff Base Nickel (II) Complexes as Potential Anticancer Drugs In Vitro
Source: Bioinorg Chem Appl. 2020 Sep 29;2020:8834859. doi: 10.1155/2020/8834859 (PMC7542481; doi:10.1155/2020/8834859)

**Supplementary Materials：**

1. **FTIR spectra of the title complexes 1, 2 and 3**

**
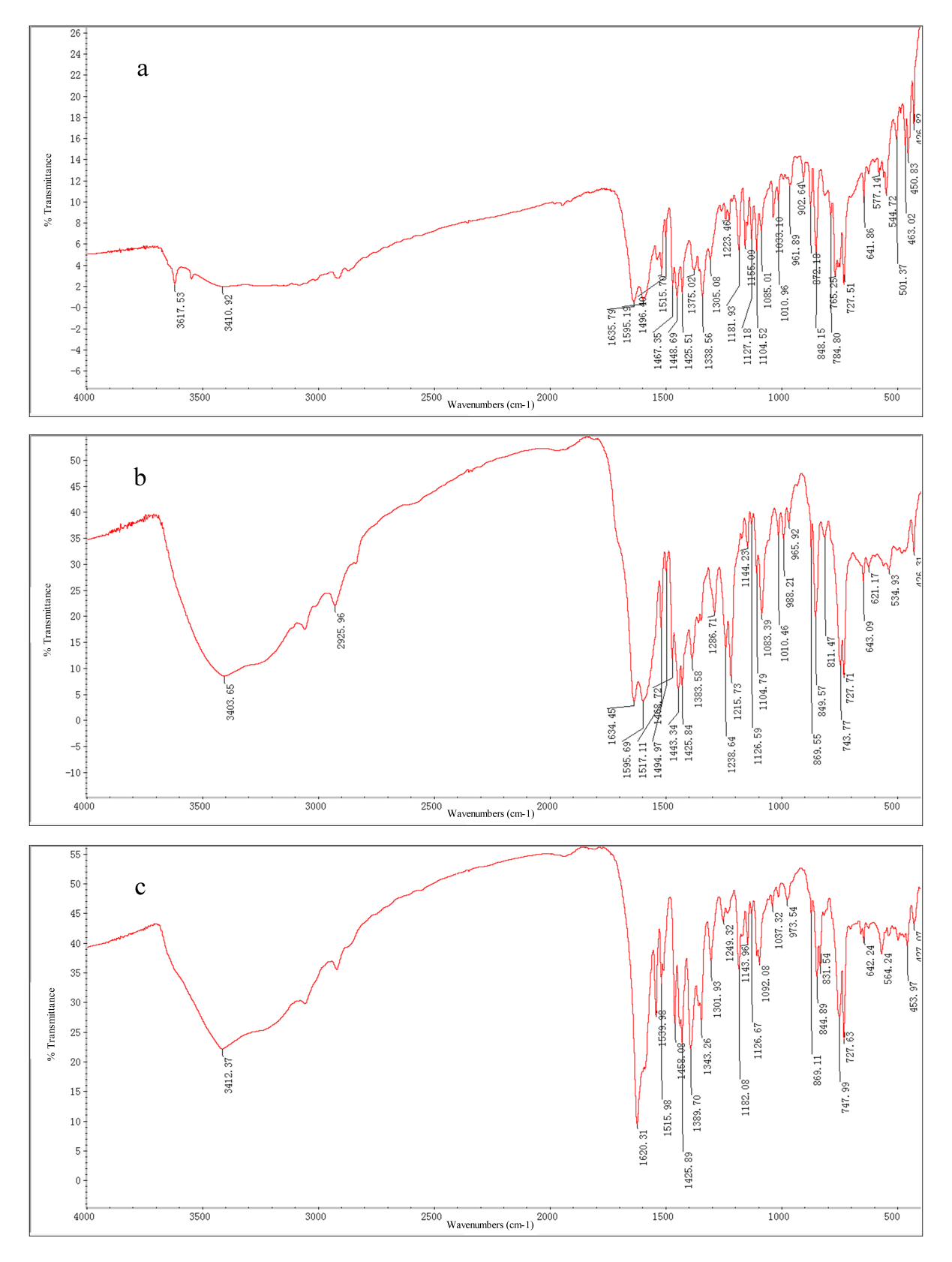
**

**Fig. S1.** FTIR spectra of the title complexes **1**, **2** and **3**.

**2. CheckCIF/PLATON reports for the crystal structure determinations of the Complexes 1, 2 and 3**

2.1 Complex **1**


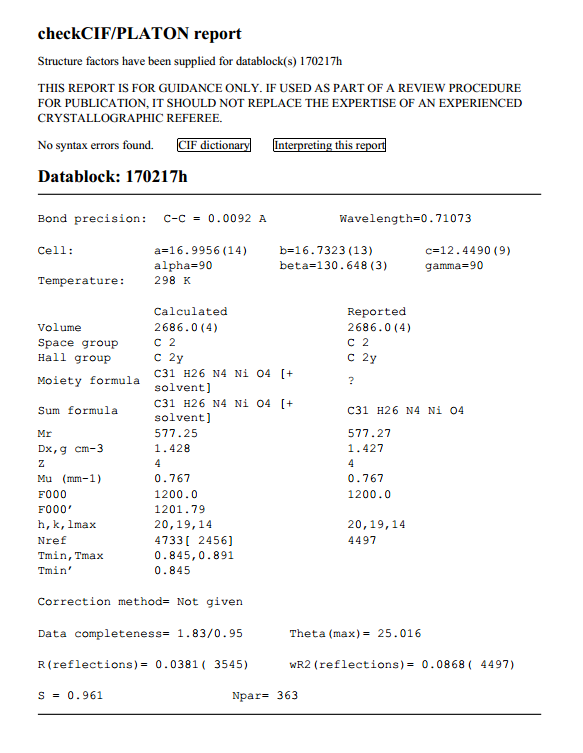

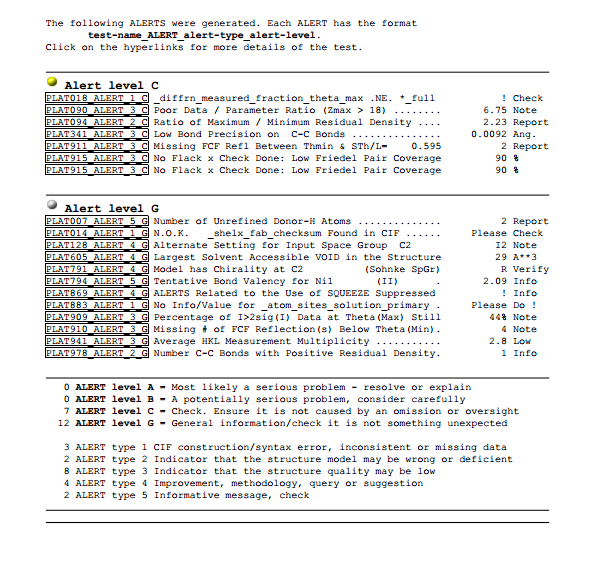

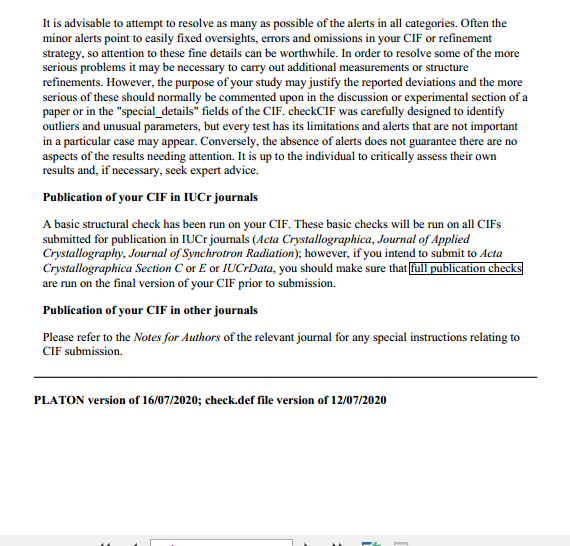

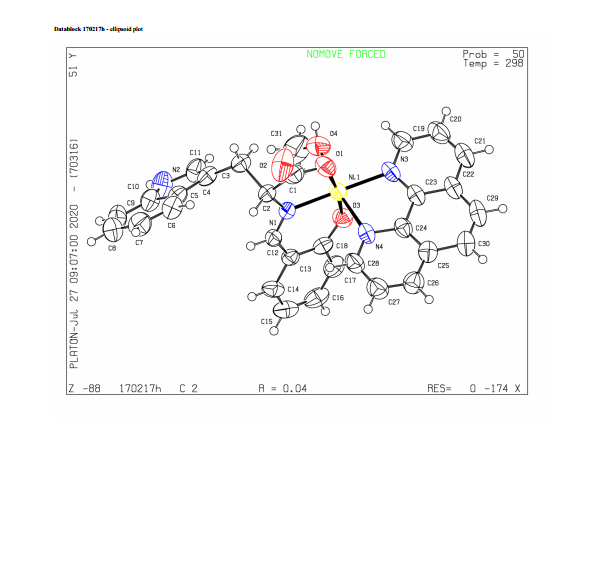


2.2 Complex **2**


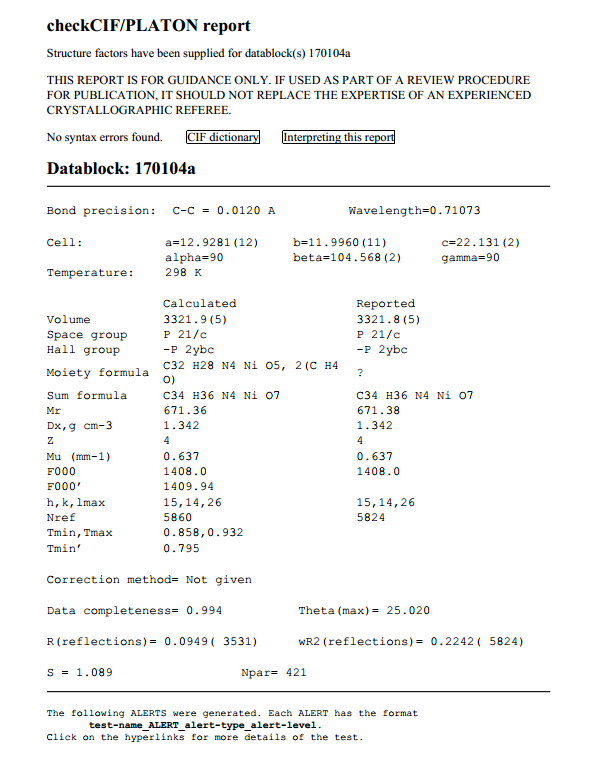


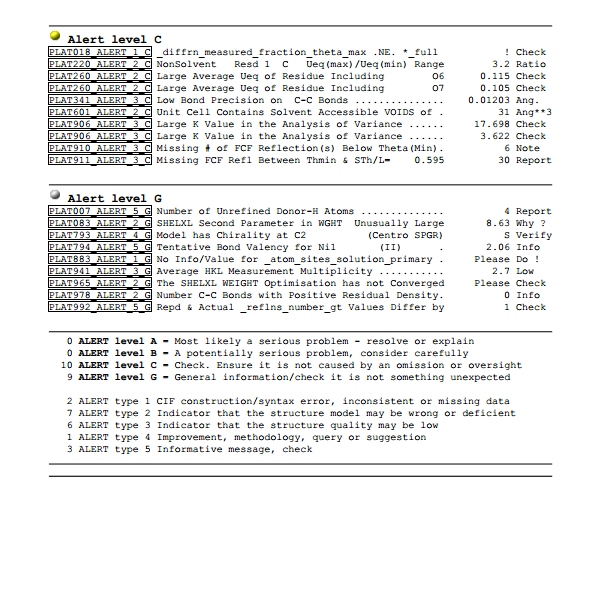

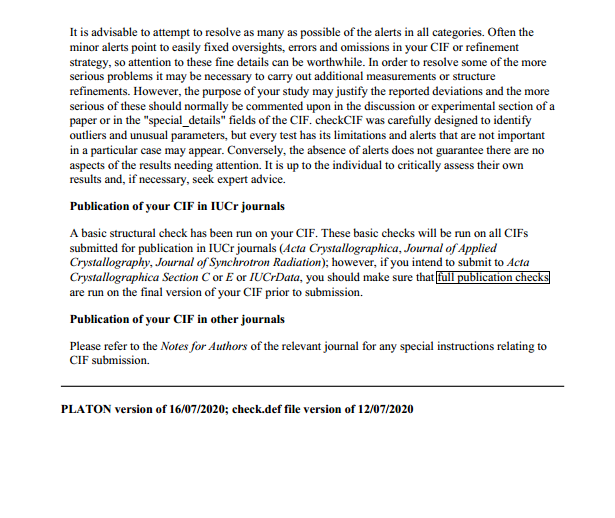

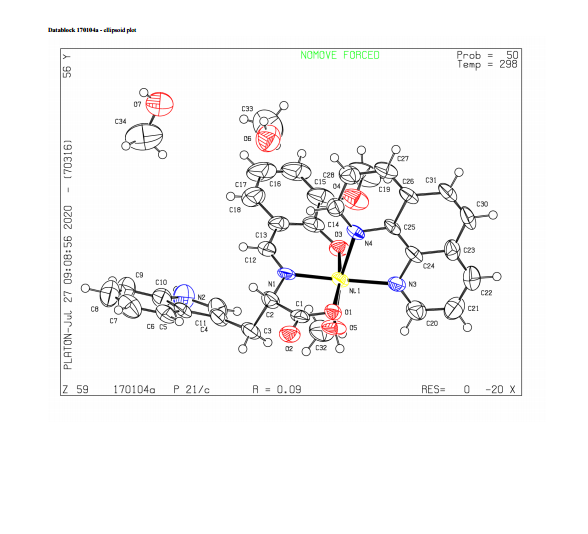


2.3 Complex **3**


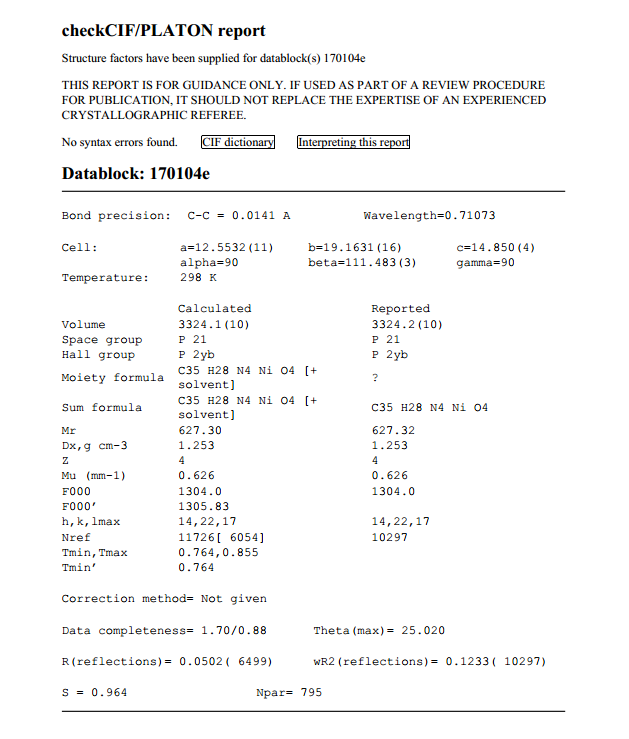


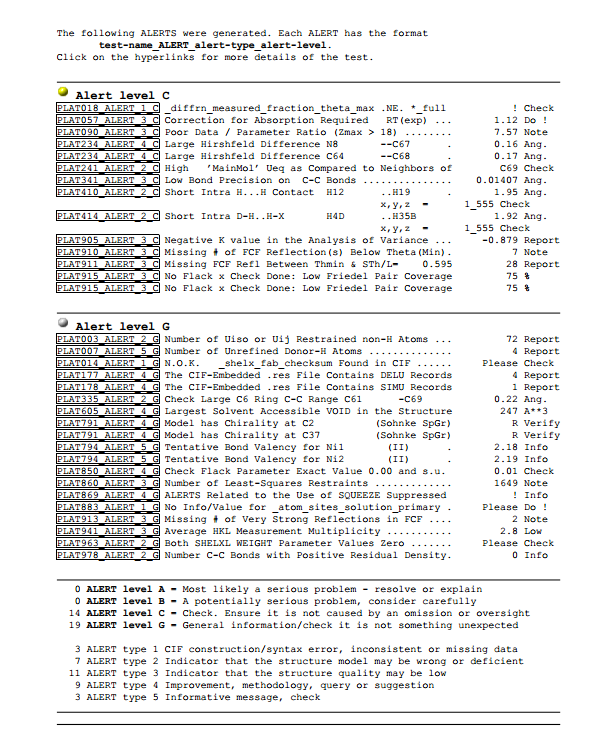


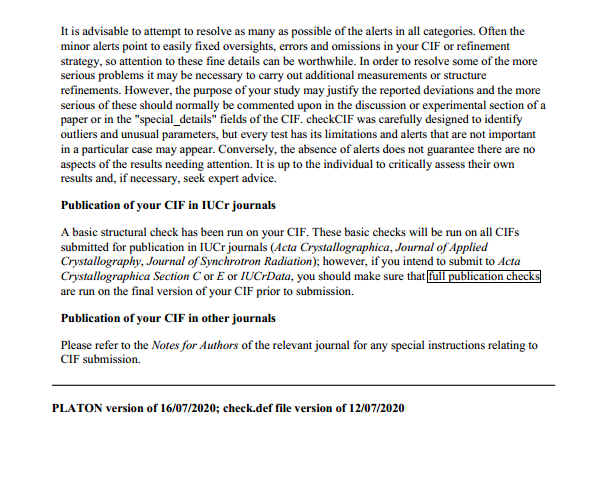


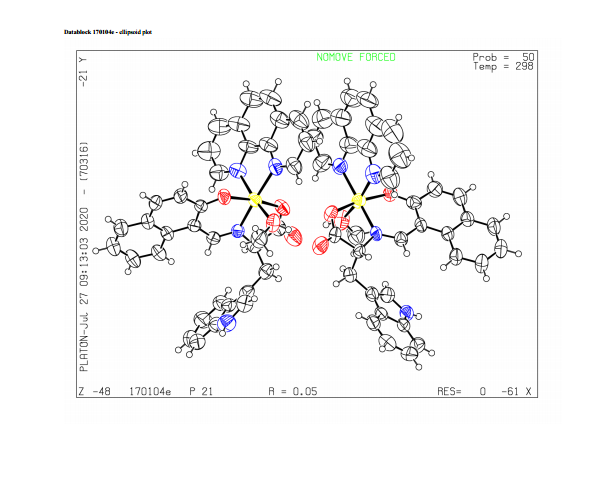

Supplement: Supplementary Materials — Figure S1: FTIR spectra of the title complexes 1, 2, and 3. Figure S2: CheckCIF/PLATON reports for the crystal structure determinations of the Complexes 1, 2, and 3. [file 8834859.f1.docx]
